# Loss of FTH1 Induces Ferritinophagy‐Mediated Ferroptosis in Anaemia of Myelodysplastic Syndromes

**DOI:** 10.1111/jcmm.70350

**Published:** 2025-01-13

**Authors:** Liyan Yang, Mengying Zhang, Mengyuan Liu, Yating Yu, Yue Zhang, Jinyue Yang, Limin Xing, Zonghong Shao, Huaquan Wang

**Affiliations:** ^1^ Department of Hematology, General Hospital Tianjin Medical University Tianjin China; ^2^ Tianjin Key Laboratory of Bone Marrow Failure and Malignant Hemopoietic Clone Control Tianjin China

**Keywords:** anaemia, ferritinophagy, ferroptosis, *FTH1*, myelodysplastic syndromes

## Abstract

Single‐cell sequencing of lineage negative (Lin‐) cells from patients with myelodysplastic syndromes (MDS) revealed a reduction in ferritin heavy chain 1 (*FTH1*) levels, yet the significance of this decrease in *FTH1* in the pathophysiology of MDS remains unclear. In this study, we evaluated the role of *FTH1* in patients with MDS. The mRNA expression of *FTH1* in GlycoA^+^ nucleated erythrocytes from MDS patients was significantly lower than that in control group. *FTH1* was implicated in both ferritinophagy and ferroptosis in MDS patients, processes that are linked to the development of anaemia. To further validate our observations, we employed shRNA to knock down the *FTH1* gene in K562 and SKM1 cells. This knockdown confirmed that the elevated ferroptosis levels observed after *FTH1* depletion were indeed due to the induction of ferritinophagy. Hemin stimulation promoted the differentiation of K562 cells, while downregulation of *FTH1* gene expression had an impact on erythroid differentiation and haemoglobin synthesis. Taken together, our results suggest that FTH1‐mediated ferritinophagy may represent a novel therapeutic target for MDS.

## 1 Introduction

Myelodysplastic syndromes (MDS) encompass a diverse group of clonal myeloid neoplasms characterised by abnormal cytogenetics, defective haematopoiesis leading to hemocytopenia, and have a risk of progressing to acute myeloid leukaemia (AML) [1]. MDS predominantly affects individuals around 70 years old, with a higher incidence among males than females, and its prevalence continues to rise annually [2]. The progression of MDS is gradual, initially presenting with anaemia as the primary symptom. However, as the disease advanced, patients typically suffer from pancytopenia, accompanied by fever, infection, anaemia, and bleeding [3].

Ineffective erythropoiesis in MDS is characterised by inadequate erythrocyte production in the bone marrow, accompanied by a notable increase in dysplastic precursor cells. Anaemia, the predominant symptom in MDS, is closely associated with this inefficiency in erythropoiesis [4]. The precise mechanism underlying ineffective erythropoiesis remains uncertain. However, our investigation into the modes of cell death in bone marrow terminally differentiated nucleated erythrocytes in MDS patients has yielded intriguing findings. Notably, we observed significant differences in the genes associated with ferroptosis compared to those in the control group. Previous studies have also suggested that ferroptosis may be the primary mechanism of decitabine‐induced MDS cell death [5].

Ferroptosis is a recently identified form of programmed cell death characterised by the accumulation of reactive oxygen species (ROS) in an iron‐dependent manner. There is mounting evidence suggesting that autophagy, the process of cellular self‐digestion, may contribute to the promotion of ferroptosis by facilitating iron accumulation or lipid peroxidation. Ferritin, a protein responsible for iron storage, can release iron through a process known as ferritinophagy, which is mediated by nuclear receptor coactivator 4 (NCOA4). This released iron forms a labile iron pool (LIP) and triggers ferroptosis. Studies have demonstrated that degradation of ferritin via ferritinophagy can enhance ferroptosis in various tumour cell lines [6, 7]. HERC2, large multi‐domain homologous to E6AP carboxy terminus (HECT) E3 ubiquitin ligase, uses its ‘CUL7‐homology domain’ to recognise NCOA4 to mediate NCOA4 turnover via the ubiquitin‐proteasome system [8]. MAP1LC3/LC3 (Microtubule‐associated protein 1 light chain 3) is the mammalian homologue of yeast ATG8 and has been used as a marker to monitor autophagy [9]; its precursor undergoes processing to remove the carboxyl terminus, producing LC3I. LC3I is then covalently conjugated to phospholipids on the autophagosome membrane through a ubiquitin‐like modification, forming LC3II. The amount of LC3II is closely related to the number of autophagosomes and is a key indicator of cellular autophagic activity. The autophagy receptor protein P62, which acts as a link between ubiquitinated substrates and LC3 located on the autophagosome membrane, can be engulfed and degraded within autophagosomes. The level of P62 protein expression is inversely proportional to autophagic activity and serves as an auxiliary indicator for assessing autophagy [10].

In addition to the accumulation of iron, ferroptosis is also characterised by a reduction in glutathione (GSH) levels and a decline in the activity of glutathione peroxidase 4 (GPX4). GPX4 is an enzyme that plays a crucial role in safeguarding cells against oxidative damage by counteracting lipid peroxides. Diminished GPX4 activity or depleted GSH levels renders the cell more vulnerable to lipid peroxidation‐induced ferroptosis [11, 12].

Iron is an essential micronutrient of all mammalian cells, acting as a cofactor in heme and proteins containing iron–sulfur clusters that regulate many biological processes, including oxygen transport and oxidative phosphorylation [13]. Cells must strike a delicate balance between iron use and storage. Fe^3+^ is absorbed by intestinal epithelial cells, binds to TF (transferrin), and circulates in the blood. Fe^3+^ enters the cell via the membrane protein TFR (transferrin receptor) and is then reduced to Fe^2+^ by STEAP3 metal‐reductase in the endosome. The release of Fe^2+^ from the endosome to the unstable iron pool in the cytoplasm requires the involvement of DMT1 (Divalent metal‐ion transporter‐1). Excess iron can be stored in ferritin or exported to the cycle by ferroportin (FPN) [14]. Ferritin, a heteromer composed of 24 subunits of ferritin heavy and light chains (FTH1, FTL), plays a crucial role in iron storage and detoxification. FTL is involved in iron nucleation, mineralization, and long‐term storage of iron within the ferritin lumen. On the other hand, the H subunit (FTH1) is responsible for rapid iron detoxification and can convert Fe^2+^ to Fe^3+^. Research has shown that the ratio of FTH1 to FTL subunits varies in different tissues. FTL‐rich ferritin is more abundant in iron‐rich organs like the liver and spleen, whereas FTH1‐rich ferritin is more prevalent in organs with low iron content, such as the heart [15]. Moreover, FTH1 has been identified as a new early erythroid precursor marker [16]. In patients with MDS, iron overload may arise as a result of factors such as ineffective erythropoiesis and repeated transfusion therapy [17]. Our research group previously conducted single‐cell RNA sequencing on lineage negative (Lin^−^) cells of MDS, after dimension reduction, clustering, and visualisation, we characterised HSC/multipotent progenitor (MPP) populations and conducted a comprehensive analysis, revealing a significant downregulation of *FTH1* expression, as well as a notable enrichment in the autophagy pathway of MDS patients [18]. FTH1 is recognised for its crucial involvement in the regulation of ferroptosis and maintenance of cellular iron homeostasis. Furthermore, it has been linked to ferritinophagy, a process characterised by the selective interaction between NCOA4 and FTH1 within autophagosomes, leading to subsequent delivery to lysosomes for iron release [19]. Nevertheless, the precise role of *FTH1* in patients with MDS and its correlation with ferritinophagy and ferroptosis have not been comprehensively investigated.

In this study, we evaluated the levels of *FTH1*, ferroptosis, and ferritinophagy‐related markers in nucleated erythrocytes during the terminal differentiation stage in the bone marrow of MDS patients. We investigated the association between *FTH1* levels and anaemia in MDS. Through knockdown experiments on the *FTH1* gene using K562 and SKM1 cell lines, we aimed to elucidate the mechanisms by which *FTH1* influences ferroptosis as well as its impact on erythroid differentiation and haemoglobin synthesis. Our findings seek to establish a theoretical foundation for enhancing ineffective erythropoiesis in MDS.

## 2 Materials and Methods

### 2.1 Patient Characteristics

The experimental cohort consisted of 73 newly diagnosed MDS patients admitted to the Haematology Department of Tianjin Medical University General Hospital from January 2021 to December 2022. These patients exhibited normal liver function, no evidence of infection, and no secondary malignancy. Among them, there were 45 male and 28 female individuals, with a median age of 64 years (range: 23–83 years). According to the 2022 WHO diagnostic classification, the cohort included 24 cases of MDS‐LB, one case of MDS‐5q, 10 cases of MDS‐SF3B1, 11 cases of IB‐1, and 27 cases of IB‐2. Karyotype analysis was performed for all patients, and 22 cases showed abnormal chromosomes. Based on the Revised International Prognostic Score System (IPSS‐R) for MDS, patients with an IPSS‐R score ≤ 3.5 were categorised as the low‐risk (LR) group, while those with an IPSS‐R score > 3.5 were designated as the high‐risk (HR) group [20]. The control group consisted of 48 cases newly diagnosed immune thrombocytopenia who had no anaemia. Table 1 presents comprehensive baseline data of the research participants. All participants in the study provided informed consent, and the research protocol was approved by the hospital ethics committee.

**TABLE 1:** Characteristics of 73 patients with myelodysplastic syndromes.

Case	Sex/Age	Diagnosis	Cytogenetics	Gene mutation	IPSS‐R
1	Male/73	MDS‐IB1	+8, ‐Y	Normal	HR
2	Male/70	MDS‐IB1	20q‐	SRSF2, KRAS, U2AF1, STAG2	HR
3	Female/61	MDS‐IB1	Normal	Normal	HR
4	Male/79	MDS‐IB1	Normal	DNMT3A, NRAS, WT1, FTL‐3TKD	HR
5	Male/73	MDS‐IB1	+8	BCOR, DNMT3A, U2AF1, SETBP1	HR
6	Male/71	MDS‐IB1	45, XY, ‐7	SRSF2, ASXL1	HR
7	Male/75	MDS‐IB1	Normal	ZRSR, TP53, IDH2	HR
8	Female/42	MDS‐IB1	Normal	U2AF1	HR
9	Male/51	MDS‐IB1	Normal	ASXL1, SETBP1, SF3B1	HR
10	Female/66	MDS‐IB1	Normal	Normal	HR
11	Female/70	MDS‐IB1	‐5, +8, 7q‐, 17p‐	Normal	HR
12	Male/56	MDS‐IB2	Normal	TP53	HR
13	Female/34	MDS‐IB2	Normal	BCOR, BCORL1, DNMT3A	HR
14	Female/64	MDS‐IB2	‐7, 5q‐	SF3B1, TP53	HR
15	Male/69	MDS‐IB2	+8	TET2, IDH2	HR
16	Male/82	MDS‐IB2	43‐44, XY, 5q‐, ‐10, ‐17, ‐18, ‐21, ‐22, 20q‐	TP53, TET2, ASXL1	HR
17	Female/80	MDS‐IB2	Normal	U2AF1, TP53	HR
18	Male/75	MDS‐IB2	7q‐	DNMT3A, JAK2, IDH1, ZRSR2, PPM1D	HR
19	Female/75	MDS‐IB2	Normal	Normal	HR
20	Male/24	MDS‐IB2	Normal	Normal	HR
21	Female/68	MDS‐IB2	5q‐, 7q‐	Normal	HR
22	Male/64	MDS‐IB2	Normal	BCOR, STAG2, ASXL1	HR
23	Female/83	MDS‐IB2	Normal	TP53	HR
24	Female/69	MDS‐IB2	Normal	ASXL1, PHF6, SRSF2, TET2	HR
25	Female/23	MDS‐IB2	Normal	NRAS, WT1	HR
26	Male/39	MDS‐IB2	Normal	Normal	HR
27	Female/41	MDS‐IB2	Normal	Normal	HR
28	Male/72	MDS‐IB2	Normal	BCOR, DNMT3A, U2AF1, BCORL1	HR
29	Female/72	MDS‐IB2	‐5, +8, 7q‐, 17p‐	TP53	HR
30	Male/65	MDS‐IB2	Normal	SRSF2, IDH1, DNMT3A, ETV6	HR
31	Male/50	MDS‐IB2	Normal	RUNX1, U2AF1	HR
32	Male/57	MDS‐IB2	Normal	RUNX1, U2AF1, BCORL1	HR
33	Male/59	MDS‐IB2	Normal	DNMT3A, SF3B1	HR
34	Male/74	MDS‐IB2	17p‐, 5q‐	TP53	HR
35	Female/51	MDS‐IB2	Normal	DDX41	HR
36	Male/66	MDS‐IB2	‐7	EZH2, NRAS, ETV6, KRAS, SETBP1	HR
37	Male/60	MDS‐IB2	Normal	DNMT3A, TP53	HR
38	Female/77	MDS‐IB2	Normal	TP53	HR
39	Male/55	MDS‐LB	+8	U2AF1, ASXL1, SETBP1	HR
40	Male/63	MDS‐LB	Normal	SRSF2, ASXL1, IDH1	LR
41	Male/33	MDS‐LB	+8	Normal	HR
42	Male/59	MDS‐LB	Normal	ASXL1, U2AF1	LR
43	Male/57	MDS‐LB	+8	RUNX1, U2AF1	HR
44	Female/64	MDS‐LB	Normal	Normal	LR
45	Female/70	MDS‐LB	Normal	Normal	LR
46	Male/70	MDS‐LB	Normal	Normal	LR
47	Female/35	MDS‐LB	Normal	Normal	HR
48	Female/63	MDS‐LB	Normal	Normal	HR
49	Female/80	MDS‐LB	Normal	Normal	LR
50	Female/38	MDS‐LB	Normal	Normal	LR
51	Male/68	MDS‐LB	Normal	Normal	LR
52	Male/69	MDS‐LB	Normal	Normal	LR
53	Male/56	MDS‐LB	5q‐, +8	P53	LR
54	Male/49	MDS‐LB	Normal	PIGA	LR
55	Female/81	MDS‐LB	Normal	ASXL1, BCOR, RUNX1	LR
56	Female/49	MDS‐LB	Normal	WT1	LR
57	Male/30	MDS‐LB	Normal	Normal	LR
58	Female/54	MDS‐LB	Normal	Normal	LR
59	Female/67	MDS‐LB	Normal	Normal	LR
60	Male/57	MDS‐SF3B1	Normal	SF3B1	LR
61	Male/67	MDS‐SF3B1	Normal	EZH2, ASXL1	LR
62	Male/55	MDS‐SF3B1	+8	DNMT3A, ZRSR2	LR
63	Male/71	MDS‐SF3B1	Normal	Normal	LR
64	Male/57	MDS‐SF3B1	20q‐, +8	Normal	HR
65	Male/68	MDS‐SF3B1	Normal	SF3B1	LR
66	Male/57	MDS‐SF3B1	Normal	U2AF1, ASXL1, ETV6	LR
67	Male/59	MDS‐SF3B1	Normal	SF3B1	LR
68	Male/78	MDS‐SF3B1	Normal	PTPN11	LR
69	Female/65	MDS‐5q‐	5q‐	Normal	LR
70	Male/60	MDS‐SF3B1	Normal	ASXL1, DNMT3A, SF3B1, TET2, RUNX1	LR
71	Male/71	MDS‐LB	20q‐	Normal	LR
72	Male/39	MDS‐LB	Normal	BCOR, CBL	LR
73	Female/72	MDS‐LB	Normal	Normal	LR

### 2.2 Reagents

CD45‐Percp, CD71‐APC, GlycoA‐PE, and GlycoA‐APC were purchased from BD Biosciences, Calcein‐acetoxymethyl ester (CAAM) is purchased from Sigma‐Aldrich, and redox‐sensitive dye BODIPY 581/591 C11 was purchased from Invitrogen Corporation. Erastin, ferrostatin‐1 (Fer‐1), and autophagy inhibitors chloroquine (CQ) were purchased from MedChemExpress. The 4‐hydroxy‐2‐nonenal (4‐HNE) ELISA kit was purchased from Elabscience and the hemin from China Topscience Corporation. GSH and malonaldehyde (MDA) assay kit was purchased from Nanjing Jiancheng Bioengineering Institute (China).

### 2.3 Cell Transfection

The shRNAs targeting *FTH1* was designed and synthesised by Genechem (Shanghai, China). The sequences of shRNAs are as follows: shRNA1 CCTGTCCATGTCTTACTACTT, shRNA2 GCTCTACGCCTCCTACGTTTA, and shRNA3 GGATATCAAGAAACCAGACTG. K562 cells were incubated with lentivirus at a multiplicity of infection (MOI) of 20, followed by the exchange of cell medium to remove residual lentivirus. The control group consisted of untreated K562 cells, while the shNC group comprised K562 cells transfected with an empty virus.

### 2.4 RNA Sequencing Analysis

RNA sequencing was performed on the BGISEQ‐500 platform (BGI‐Shenzhen, China) using RNA extracted from 6 cell samples, comprising 3 from the shFTH1 group and 3 from the shNC group. Data visualisation was achieved using the Dr.Tom system provided by BGI. Random sampling was employed during RNA sequencing. To ensure the accuracy of the differentially expressed gene analysis, genes were considered differentially expressed when the fold change was ≥ 1 and the *Q* value was ≤ 0.05.

### 2.5 Flow Cytometry (FCM)

Bone marrow samples were obtained from both MDS patients and controls using heparin anticoagulant tubes. Nucleated erythrocytes were subsequently labelled with Percp‐anti‐CD45, PE‐anti‐GlycoA, and APC‐anti‐CD71. The CAAM method was used to assess LIP. As calcein fluorescence is quenched upon chelating labile iron, the degree of quenching correlates with the cellular LIP [21]. Lipid peroxidation was measured by flow cytometry using C11‐BODIPY dye that changes its fluorescence emission from red to green upon oxidation. The samples were incubated with 0.125 μM CAAM and 10 μM C11‐BODIPY at 37°C for 30 min. Subsequently, more than 30,000 cells were acquired using a FACS Calibur flow cytometer (Beckman Coulter) and analysed using CytExpert software version 3.1 (BD Biosciences, USA).

### 2.6 Real‐Time Quantitative Transcriptase‐Polymerase Chain Reaction (qRT‐PCR)

GlycoA^+^ nucleated erythrocytes were sorted by GlycoA microbeads (Bruker, Germany). Total RNA was extracted using TRIzol (Takara Bio USA Inc.), and cDNA was generated using a reverse transcriptase kit (Takara Bio USA Inc.). The gene expressions were quantified by Q‐PCR (SYBR Premix Ex Taq II, Takara Bio, China). The primer sequences were as follows: *FTH1* forward 5′‐CCAGAACTACCACCAGGACTC‐3′, reverse 5′‐GAAGATTCGGCCACCTCGTT‐3′; *FTL* forward 5′‐TGGAGACTCACTTCCT AGATGA‐3′, reverse 5′‐TGAGCCTTTCGAAGAGATACTC‐3′; *NCOA4* forward 5′‐TGTGAATGATTGGCTTGTCAAG‐3′, reverse 5′‐CACACCTCCTCTACCTTA CATG‐3′, *LC3B* forward 5′‐TTCAGGTTCACAAAACCCGC‐3′, reverse 5′‐TCTC ACACAGCCCGTTTACC‐3′; *HERC2* forward 5′‐GGAGCGAGATCCCTCCAAAT‐3′, reverse 5′‐GGCTGTTGTCATACTTCTCATGG‐3′; *TFR* forward 5′‐GAGCGTC GGGATATCGGGT‐3′, reverse 5′‐CAGGATGAAGGGAGGACACG‐3′; *FPN* forward 5′‐TGAGCCTCCCAAACCGCTTCCATA‐3′, reverse 5′‐GGGCAAAAAGACTAC AACGACGACTT‐3′; *DMT1* forward 5′‐AGCCACTCAGGTATCCACCAT‐3′, reverse 5′‐CCAGGGGACTATGAAAGAGAG‐3′; *GAPDH* forward 5′‐CAGGAGG CATTGCTGATGAT‐3′, reverse 5′‐GAAGGCTGGGGCTCATTT‐3′; To generate the relative quantification (RQ) of the gene expression, the 2^−ΔΔCt^ method was used: ΔΔCt = Ct_target_ − Ct_GAPDH_ patients − Ct_target_ − Ct_GAPDH_ controls.

### 2.7 ELISA

Bone marrow samples were obtained from MDS patients and controls using heparin anticoagulant tubes. The concentration of 4HNE in bone marrow plasma was quantified by ELISA.

### 2.8 Western Blot

The total proteins of K562 cells were extracted by lysis buffer (Biotech, Beijing, China). Protein concentration was determined using the Pierce BCA Protein Assay Kit (Thermo Scientific, USA). Samples were separated by 4%–20% SDS‐PAGE gels and then transferred to PVDF membranes (PE, USA) by a Trans‐Blot Cell system (Bio‐Rad, USA) using standard western blotting procedures. The membranes were probed with a rabbit anti‐human FTH1/NCOA4/ACSL4/Beclin1/LC3B (Cell Signalling Technology, USA)/4‐HNE antibody (Invitrogen, USA) at 1:1000 and incubated overnight at 4°C. The rabbit anti‐human *β*‐actin antibody (Santa Cruz Biotechnology Inc., sc‐47,778, USA) at 1:5000 was used as loading control. The goat anti‐rabbit IgG HRP‐conjugated secondary antibody (Santa Cruz Biotechnology Inc., sc‐2054, USA) was incubated for 1 h at room temperature. After washing, chemiluminescence was detected using an electrochemiluminescence (ECL) reagent from Thermo Scientific (number 32106, USA).

### 2.9 Cell Viability Assay

All cells were cultured in 96‐well plates (2 × 10^4^ cells/mL) and were treated with Fer‐1 (10 μM), erastin (10 μM), and CQ (1 μM) for 24 h. Subsequently, 10 μL CCK‐8 reagent was added to the wells, and the cells were incubated for 2 h at 37°C. The absorbance values were measured at 450 nm using a Bio‐Rad Model 450 microplate reader (Bio‐Rad, CA, USA).

### 2.10 GSH And MDA Determination

The cells were collected and washed twice with phosphate‐buffered saline (PBS). Subsequently, PBS was added and the cells were then subjected to ultrasonic disruption. The cell suspension after ultrasonic crushing was taken and operated according to manufacturer's instruction. The absorbance at 405 nm/450 nm was detected by the microplate reader. GSH/MDA content was normalised to protein concentration and expressed as μM per mg protein.

### 2.11 Quantification of Iron Levels

The iron assay kit (Biosciences) was utilised for the quantification of iron levels in K562 cells (1 × 10^7^). The cells were harvested and washed with cold PBS, followed by homogenization in 200 μL of iron assay buffer using ultrasound. Subsequently, the samples underwent centrifugation at 10,000 ***g*** for 10 min to eliminate insoluble materials. A volume of 100 μL of assay buffer was then added to each well for the measurement of Fe^2+^ levels, with absorbance readings taken at 510 nm using a microplate reader.

### 2.12 Benzidine Staining

Hemin (40 μm) was used to induce differentiation of K562 cells for four consecutive days, with induced cells being subjected to daily benzidine staining. The presence of haemoglobin production was observed under a light microscope, resulting in the identification of benzidine positive cells stained light blue.

### 2.13 Statistical Analysis

GraphPad Prism8 was employed for data visualisation, and SPSS 22.0 was used for data analysis. For normally distributed data, data were presented as mean ± standard deviation; two‐tailed unpaired Student's *t* test was used for comparing two groups of data, and one‐way analysis of variance (ANOVA) was used for multiple group comparisons. In the case of non‐normally distributed data, data were presented as the median and range; the Mann–Whitney test was used for two‐group comparisons, and the Kruskal‐Wallis test was used for multiple‐group comparisons. Pearson correlation analysis was used for normally distributed data, while Spearman correlation analysis was applied for non‐normally distributed data. A significance level of *p* ≤ 0.05 was considered statistically significant in all analyses.

## 3 Results

### 3.1 The Ferroptosis Levels in Terminally Nucleated Red Blood Cells From the Bone Marrow of MDS Patients Were Found to Be Elevated

**FIGURE 1:**
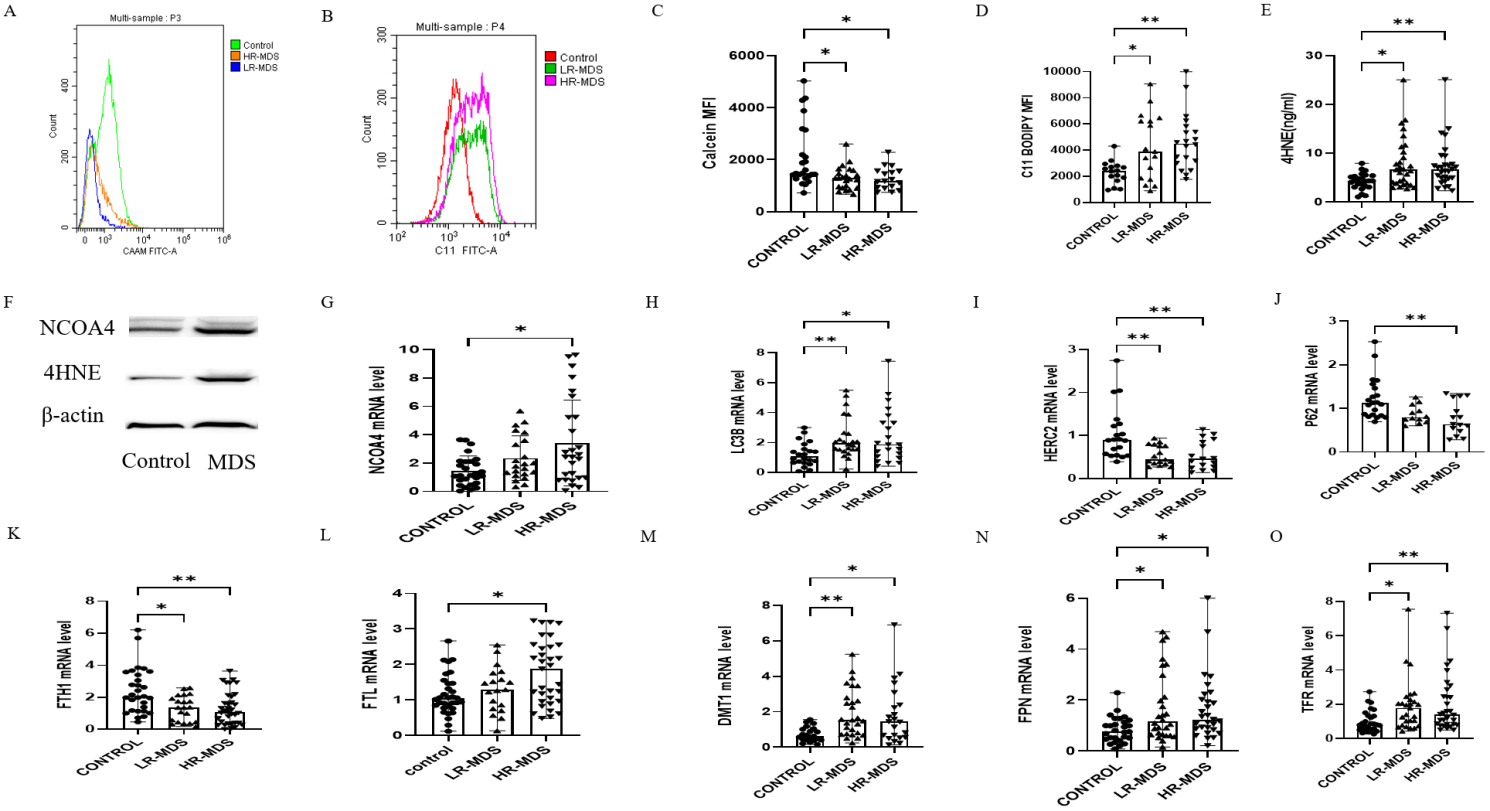
The levels of ferroptosis and ferritinophagy in bone marrow terminally differentiated nucleated erythrocytes increased in MDS patients. (A) Histograms indicating the difference in calcein fluorescence intensity between MDS patients and the control group. (B) Histograms indicating the fluorescence level of intracellular oxidised BODIPY in MDS groups and control. (C) The bar chart shows the quantitative analysis of calcein mean fluorescence intensity (MFI) being significantly lower in the MDS group. (D) The bar chart shows the quantitative analysis of C11‐BODIPY MFI (significantly higher in MDS patients). (E) Differences in the levels of 4‐hydroxynonenal (4‐HNE) lipid peroxidation marker, measured by ELISA in the bone marrow plasma of MDS and control groups. (F) A representative Western blot image of NCOA4 and 4HNE protein levels in MDS and control groups, β‐Actin being used as a loading control. (G–O) Comparison of *NCOA4* (G), Light chain 3B (*LC3B*) (H), *HERC2* (I), *P62*mRNA levels (J), iron homeostasis gene (*FTH1*, *FTL*, *DMT1*, *FPN*, and *TFR*) mRNA levels (K–O) of GlycoA^+^ nucleated erythrocytes in MDS patients and control group. Data are presented as mean ± SD for (G). Data were presented as (median range) for (C–E, H–O). The Kruskal‐Wallis test for (C–E) and (G–O). *p < 0.05, **p < 0.01.

Anaemia is the predominant manifestation of MDS and is linked to ineffective erythropoiesis. The involvement of ferroptosis in the pathogenesis of ineffective haematopoiesis in MDS remains uncertain. Hence, we assessed the levels of ferroptosis in terminally differentiated nucleated erythrocytes from the bone marrow of MDS patients. Iron and lipid peroxidation levels were used as necessary indicators of ferroptosis. The levels of the LIP and lipid peroxidation in bone marrow terminally differentiated nucleated erythrocytes were significantly higher in the MDS group compared to the control group (Figure 1A–D). Lipid peroxidation, a critical process in ferroptosis [22], leads to the generation of MDA and 4‐HNE. The expression level of 4‐HNE in the MDS group was significantly higher than that in the control group (Figure 1E,F). All the above results showed that the levels of ferroptosis of terminally differentiated nucleated erythrocytes in bone marrow were elevated in MDS patients.

### 3.2 The Levels of Ferritinophagy in Terminally Nucleated Red Blood Cells From the Bone Marrow of MDS Patients Were Elevated

Inducing ferroptosis led to the activation of autophagy and subsequent degradation of the ferritinophagy cargo receptor, NCOA4 [6]. We also evaluated the levels of ferritinophagy in terminally differentiated nucleated erythrocytes from the bone marrow of MDS patients. The purity of GlycoA^+^ nucleated erythrocytes exceeded 97% (Figure S1). LC3B serves as a reliable indicator for the process of autophagy. The levels of *NCOA4* and *LC3B* in GlycoA^+^ nucleated erythrocytes from the MDS group were significantly higher than those in the control group (Figure 1F–H). The level of P62 was found to be inversely correlated with the level of autophagy. Additionally, it was observed that HERC‐2 plays a role in the degradation of NCOA4. The mRNA expression levels of *HERC2* and *P62* in GlycoA^+^ nucleated erythrocytes from the MDS group were notably lower compared to those in the control group (Figure 1I,J). A decrease in *P62* and *HERC2*, along with increases in *LC3B* and *NCOA4*, indicates enhanced ferritinophagy.

### 3.3 The Iron Homeostasis Levels in Terminally Nucleated Red Blood Cells From the Bone Marrow of MDS Patients

As a result, we conducted an in‐depth analysis of key cellular iron homeostasis genes. *FTH1*, known for its pivotal role in both ferroptosis and the maintenance of cellular iron balance, exhibited significantly decreased mRNA expressions in the MDS group compared to the control group. Notably, the *FTL* mRNA levels in the HR‐MDS group were markedly elevated compared to both the control and LR‐MDS group (Figure 1K,L).

*TFR*, which regulates the cellular uptake of iron via Fe^3+^‐transferrin, was significantly elevated in the MDS group compared to the control group. Moreover, there was an observed increase in the *DMT1*, responsible for transporting Fe^2+^ from endosomes to the cytoplasm. Additionally, *FPN*, involved in effluxing iron from the cell, exhibited a significant upregulation (Figure 1M–O).

### 3.4 Elevated Ferroptosis and Increased Ferritinophagy Levels in the Bone Marrow of Patients With MDS Were Correlated With the Presence of Anaemia

**FIGURE 2:**
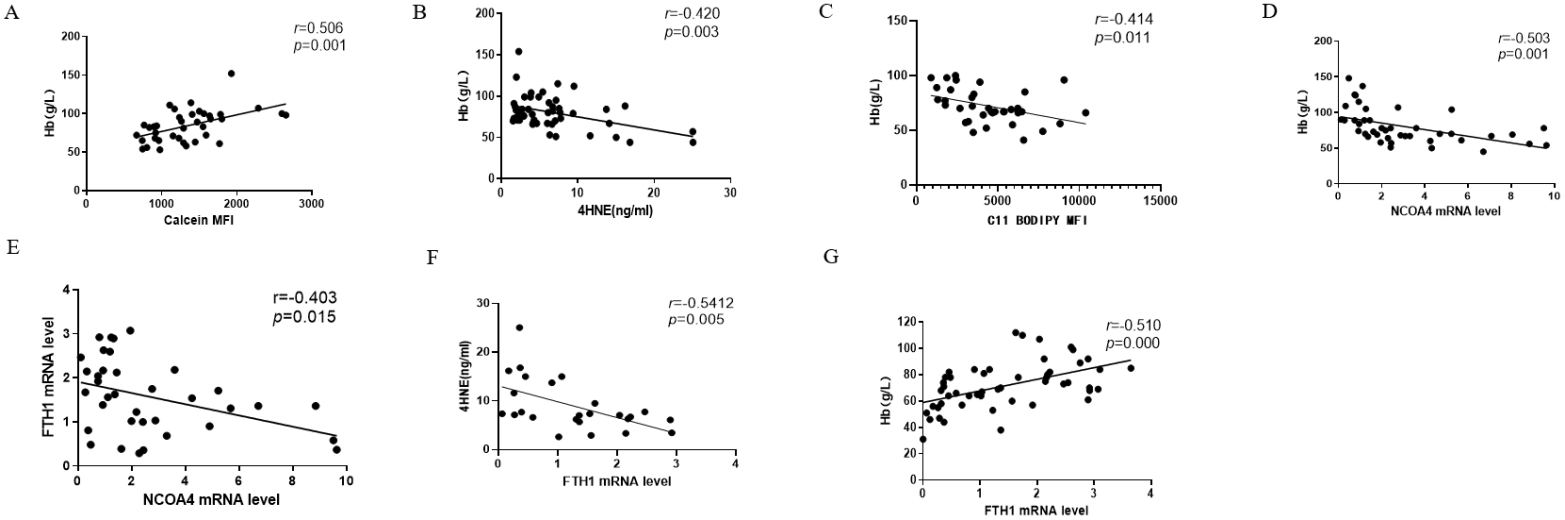
Increased ferroptosis and ferritinophagy levels in bone marrow terminally differentiated nucleated erythrocytes were associated with anaemia in MDS patients. (A) Correlation analysis between calcein mean fluorescence intensity (MFI) and haemoglobin (Hb) concentration. (B) Correlation analysis between 4HNE concentration and Hb concentration. (C) Correlation analysis of C11‐BODIPY MFI and Hb concentration. (D, E) Correlation analysis of *NCOA4* mRNA levels and Hb concentration (D), *FTH1* mRNA levels (E). (F, G) Correlation analysis of *FTH1* mRNA levels and 4HNE concentration (F), Hb concentration (G).

We investigated the potential association between levels of ferritinophagy and ferroptosis with the observed anaemia in MDS. Our correlation analysis revealed a negative relationship between LIP and lipid peroxidation and haemoglobin (Hb) concentration in bone marrow nucleated erythrocytes of MDS patients. Furthermore, we found that the concentration of 4‐HNE in the bone marrow plasma of MDS patients was negatively correlated with Hb concentration (Figure 2A–C). These results suggested a link between heightened levels of ferroptosis in terminally differentiated nucleated erythrocytes within the bone marrow of MDS patients and the occurrence of anaemia. The *NCOA4* mRNA levels in GlycoA+ nucleated erythrocytes of MDS patients exhibited a negative correlation with Hb concentration (Figure 2D) and a positive correlation with mean corpuscular volume (MCV) and the percentage of reticulocytes (Ret) (Figure S2A,B). These results strongly suggested that the increased ferritinophagy levels in terminally differentiated nucleated erythrocytes within the bone marrow of MDS patients are associated with the occurrence of anaemia.

### 3.5 The Expression Level of *FTH1* in Bone Marrow Nucleated Red Cells of Patients With MDS Was Found to Be Significantly Associated With *NCOA4* , 4HNE, and the Severity of Anaemia

Reduced levels of FTL during pregnancy may initiate ferroptosis subsequently resulting in impaired remodelling of the uterine spiral arteries and ultimately leading to the development of preeclampsia [23]. Did the involvement of *FTH1* or *FTL* in ferritinophagy occur? Our findings indicate a negative correlation between the expression of *FTH1* mRNA and *NCOA4* in GlycoA+ nucleated erythrocytes of MDS patients (Figure 2E). Conversely, there was no discernible correlation between the expression of *NCOA4* and *FTL* in GlycoA+ nucleated erythrocytes of MDS patients (Figure S2C). Hence, we hypothesize that FTH1 plays a role in the process of ferritinophagy. Consequently, we sought to investigate whether alterations in *FTH1* levels impact the degree of ferroptosis contributing to MDS‐related anaemia. Subsequently, we conducted an analysis to assess the relationship between *FTH1* expression and 4HNE and Hb. The expression of *FTH1* mRNA in GlycoA+ nucleated erythrocytes of MDS patients exhibited a negative correlation with the concentration of 4‐HNE (Figure 2F) and a positive correlation with the concentration of Hb (Figure 2G), MCV, and the percentage of Ret in MDS patients (Figure S2D,E). Interestingly, the expressions of *FTH1* mRNA in GlycoA+ nucleated erythrocytes weakly negatively correlated with absolute neutrophil counts (ANC) in MDS patients. However, no significant correlation was observed between the levels of *FTH1* in GlycoA+ nucleated erythrocytes and the platelet counts (PLT) in MDS patients (Figure S2F,G). Finally, the expression of *FTH1* mRNA in GlycoA+ nucleated erythrocytes was negatively correlated with the concentration of ferritin in MDS patients (Figure S2H). These findings provide evidence that *FTH1* is intricately involved in ferritinophagy and ferroptosis within the bone marrow nucleated erythrocytes of MDS patients and further supports its association with anaemia.

### 3.6 Differential mRNA Expression Profile Following FTH1 Knockdown

To investigate the regulatory role of *FTH1*, we employed lentivirus transfection to knock down the *FTH1* gene in K562 and SKM1 cells, achieving a knockdown efficiency of over 95% (Figure S3). Subsequently, we knocked down the *FTH1* gene in K562 cells and analysed the results through RNA sequencing. The top KEGG pathway analysis revealed that these differentially expressed genes were significantly enriched in processes including cell cycle regulation, cell senescence, ferroptosis, mitochondrial autophagy, and lysosomal pathways. In terms of biological processes, the differential genes were predominantly associated with GO terms such as cell division, cell cycle control, negative regulation of apoptosis, and oxygen transport, among others. When considering molecular functions, GO terms related to RNA binding, protein binding, globin binding, and haemoglobin A binding were prevalent. Furthermore, in terms of cellular components, the cytoplasm, nucleus, and haemoglobin complex were among the prominently enriched GO terms. Notably, functions related to oxygen transport, globin binding, haemoglobin A binding, and haemoglobin complex are directly linked to haemoglobin synthesis. This suggested that FTH1 exerts an influence on both cell proliferation and cell death, which is intricately tied to the manifestation of anaemia.

### 3.7 Knockdown of FTH1 Resulted in Cell Ferroptosis

**FIGURE 3:**
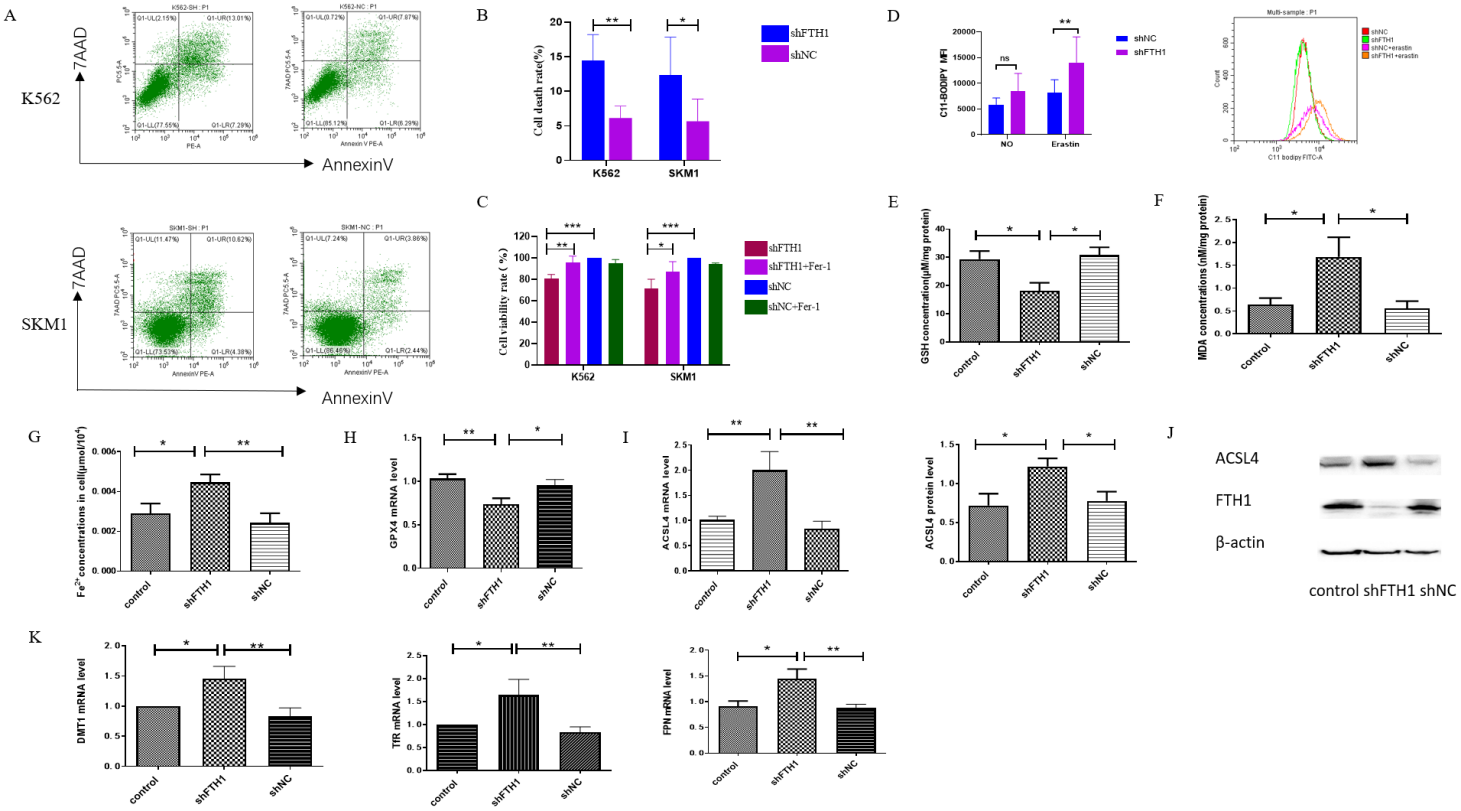
The levels of ferroptosis increased after FTH1 knockdown in K562 and SKM1 cells. (A) Cell death induced by FTH1 knockdown in K562 and SKM‐1 cells revealed by flow cytometry. (B) The corresponding bar chart depicting quantitative analysis of cell death rates between the shFTH1 and shNC group in K562 and SKM‐1 cells. (C) Comparison of cell viability rates in shNC, shNC + Fer‐1, shFTH1, shFTH1 + Fer‐1 groups in K562 and SKM‐1 cells. (D) Comparison of C11‐BODIPY mean fluorescence intensity (MFI) in shFTH1, shNC, shFTH1 + erastin and shNC + erastin groups. (E–I) Comparison of GSH (E), MDA (F), Fe^2+^ content (G), *GPX4* mRNA levels (H), *ACSL4* mRNA and protein levels (I) in shFTH1, shNC, control groups. (J) A representative Western blot image of ACSL4 and FTH1 protein levels in shFTH1, shNC and control groups, β‐Actin being used as a loading control. (K) The level of iron homeostasis gene (*DMT1*, *TFR*, and *FPN*) after FTH1 knockdown in K562 cells. Control, Untreated K562 cells. shNC, K562 cells transfected with empty virus. Data were presented as mean ± SD. Two‐tailed unpaired Student's *t* test for (B–D, E, G, H) and last panel in (I). Mann–Whitney test for (F), (K), and first panel in (I). *p < 0.05, **p < 0.01, ***p < 0.001.

Subsequently, we conducted an assessment and found that the proportion of cell death in the shFTH1 group was significantly higher (Figure 3A,B). To further substantiate that the mode of cell death following *FTH1* knockdown was attributed to the ferroptosis pathway, we applied Fer‐1 to inhibit ferroptosis. As a result, the cell viability rates in the shFTH1 treated group were markedly higher than those in the untreated shFTH1 group (Figure 3C). Furthermore, we measured the levels of Fe^2+^, the expression of *GPX4*, lipid peroxidation, GSH, and MDA as indicators of ferroptosis. Upon treatment of K562 cells with erastin, the lipid peroxidation levels in the shFTH1 + erastin group were significantly elevated (Figure 3D). Additionally, we observed an increase in MDA concentration and iron levels, along with a decrease in *GPX4* mRNA levels and GSH content in the shFTH1 group when compared to the control (Figure 3E–H). The expression of ACSL4 has been linked to the sensitivity of cancer cells to ferroptosis. Notably, the expression of ACSL4 in the shFTH1 group was significantly higher than the control group (Figure 3I,J).

Following *FTH1* knockdown in K562 cells, we assessed the levels of iron homeostasis. The mRNA levels of *DMT1*, *FPN*, and *TFR* in the shFTH1 group were notably higher than those in both the shNC and control groups (Figure 3K).

### 3.8 Knockdown of FTH1 Resulted in Cell Ferroptosis Through the Process of Ferritinophagy

**FIGURE 4:**
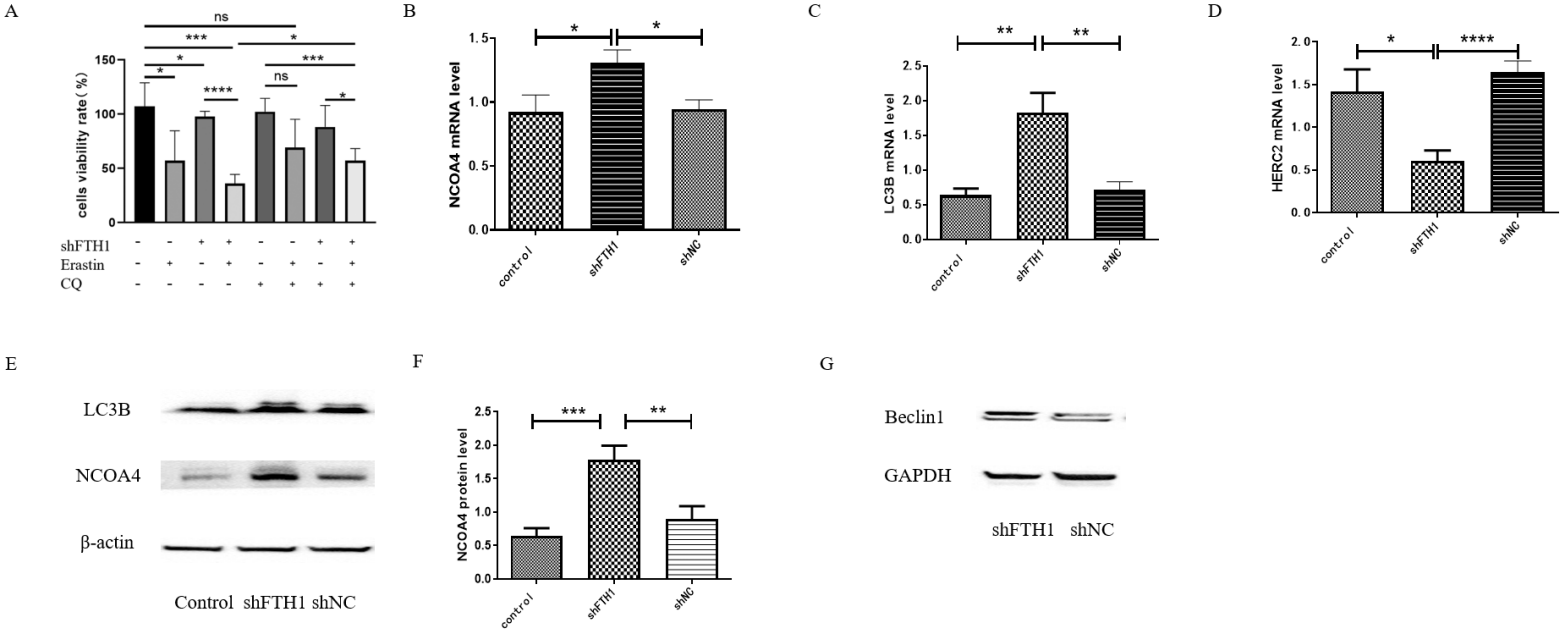
The knockdown of FTH1 resulted in increased ferroptosis level due to induce ferritinophagy in K562 cells. (A) Cell viability rates in erastin, CQ treatment control group and shFTH1 group. (B–D) Comparison of *NCOA4*, *LC3B* and *HERC2* mRNA levels in K562 cells from the control, shNC, and shFTH1groups. (E, F) A representative Western blot image of NCOA4 and LC3B (E) protein levels in shFTH1, shNC and control groups, β‐actin being used as a loading control, and the bar charts indicate the quantitative analysis of NCOA4 (F) in K562 cells from the control and shFTH1groups. (G) A representative Western blot image of Beclin1 protein levels in shFTH1and shNC groups, GAPDH being used as a loading control. Control, Untreated K562 cells. shNC, K562 cells transfected with empty virus. Data were presented as mean ± SD. Two‐tailed unpaired Student's *t* test for (A, B, D, F). Mann–Whitney test for (C). *p < 0.05, **p < 0.01, ***p < 0.001, ****p < 0.0001.

The above results validate the pivotal role of *FTH1* in ferroptosis. As *FTH1* is also a central player in ferritinophagy, we subsequently delved into the regulatory mechanism of *FTH1* in this process. We observed that the levels of NCOA4 and LC3B in the shFTH1 group were significantly higher compared to both the control and shNC groups (Figure 4B,C,E,F). Moreover, the *HERC2* mRNA level in the shFTH1 group was notably lower than that in both the control and shNC groups (Figure 4D). Autophagosome formation necessitates the involvement of beclin1, which enhances the induction of autophagy. Western blot results revealed an increase in the beclin1 protein levels in cells of the shFTH1 group (Figure 4G). To further investigate, we induced ferroptosis using erastin. Additionally, we utilised CQ to inhibit autophagic flux. Interestingly, the cell viability rate in the shFTH1 CQ treatment group was significantly higher than that in the shFTH1 erastin+CQ treatment group and notably lower than that in the shFTH1 erastin group. This strongly suggests that the induction of ferritinophagy was heightened after *FTH1* knockdown, and subsequently, cell viability rates increased following the inhibition of autophagy by CQ (Figure 4A).

### 3.9 Knockdown of FTH1 Had a Significant Impact on the Differentiation of Erythroid Cells and the Synthesis of Haemoglobin in K562 Cells

**FIGURE 5:**
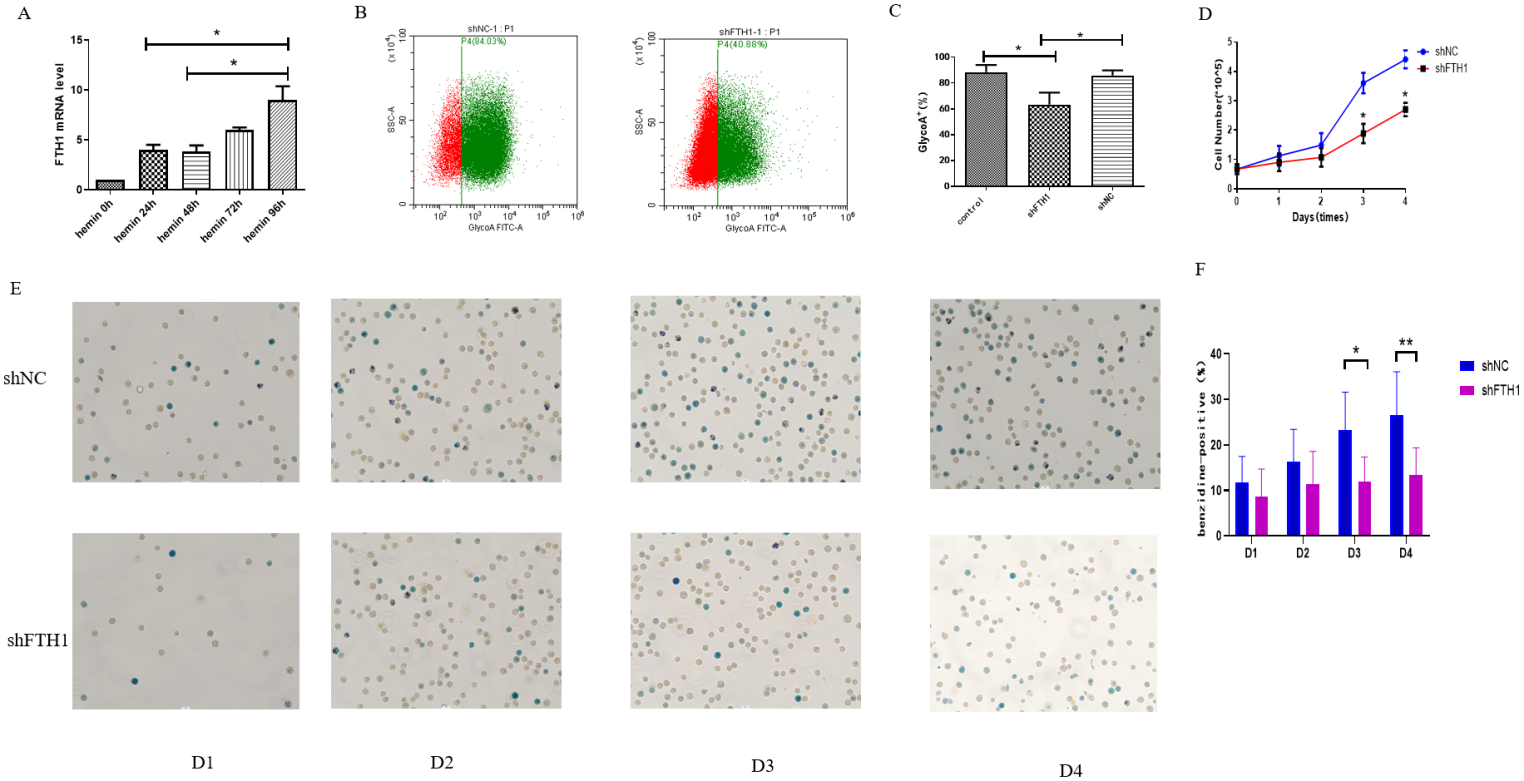
Knockdown of FTH1 had a significant impact on the differentiation of erythroid cells and the synthesis of haemoglobin in K562 cells. (A) Quantification of *FTH1* mRNA expression levels in K562 cells treated with hemin for 4 consecutive days. (B, C) Flow cytometry analysis of GlycoA‐FITC labelled GlycoA expression and the corresponding bar chart depicting quantitative analysis of GlycoA expression rates in control, shNC, and shFTH1 groups. (D) Growth curves comparing shNC and shFTH1 groups following hemin induction. (E, F) Assessment of haemoglobin synthesis in K562 cells following hemin induction in the shNC and shFTH1 groups. Control, Untreated K562 cells. shNC, K562 cells transfected with empty virus. Data were presented as mean ± SD. Two‐tailed unpaired Student's *t* test for (A, D, F). Mann–Whitney test for (C). *p < 0.05, **p < 0.01.

To assess the influence of FTH1 knockdown on erythroid differentiation in K562 cells, we initially validated the presence of *FTH1* gene expression in K562 cells. Notably, the mRNA expression levels of *FTH1* exhibited a gradual increase from day one to day four following hemin treatment (Figure 5A). GlycoA serves as a marker for late‐stage erythroid differentiation. We observed that GlycoA expression was significantly lower in the shFTH1 group compared to both the control and shNC groups (Figure 5B,C). Furthermore, upon induction of differentiation with hemin, the number of cells in the *FTH1* knockdown group was notably lower than that in the control group (Figure 5D).

The erythroid hemoglobinogenesis in K562 cells was assessed using benzidine staining, and the percentage of benzidine‐positive cells was documented. Interestingly, there were no significant differences between the shFTH1 group and the shNC group on day 1 and day 2. However, it is noteworthy that the differentiation process in the shFTH1 group exhibited a slower pace compared to the control group on day 3 (11.99 ± 5.44 vs. 22.01 ± 9.12) and day 4 (13.42 ± 5.91 vs. 26.63 ± 9.46) (Figure 5E,F).

## 4 Discussion

Although FTH1 is known to play a crucial role in ferroptosis [24], its role in ineffective haematopoiesis in MDS remains unclear. In this study, we demonstrated that decreased levels of FTH1 are associated with increased ferroptosis and ferritinophagy. Specifically, *FTH1* participates in ferritinophagy and ferroptosis and is linked to anaemia in GlycoA+ nucleated erythrocytes of MDS patients. We further investigated the impact of FTH1 by knocking down its gene in K562 and SKM1 cell lines. Our results showed that FTH1 knockdown led to an increase in ferroptosis, which was attributed to the induction of autophagy. Additionally, FTH1 knockdown affected erythroid differentiation and haemoglobin synthesis. This study offers new insights into the treatment of MDS‐related anaemia by highlighting the potential therapeutic value of targeting FTH1.

Our results showed no statistically significant differences in the indicators of ferroptosis and ferritinophagy between LR‐MDS and HR‐MDS patients. However, there were statistically significant differences when comparing LR‐MDS patients with the control group and HR‐MDS patients with the control group, suggesting that the levels of ferritinophagy and ferroptosis are elevated in MDS patients, regardless of whether they are low‐risk or high‐risk. This suggests that ferroptosis and ferritinophagy may play an important role in the pathological process of MDS.

Ferritinophagy is the autophagic degradation of ferritin to maintain homeostasis during iron consumption. Ferritinophagy plays a crucial role in various diseases. In mice, sorafenib alleviates liver fibrosis by inducing ferroptosis in hepatic stellate cells. Subsequent studies have demonstrated that ferritinophagy is necessary for ferroptosis to inhibit liver fibrosis [25, 26]. Pancreatic ductal cancer relies on both autophagy and iron metabolism, yet there are currently no targeted therapies addressing these pathways. Ferritinophagy supports iron metabolism in pancreatic ductal cancer, thereby promoting tumour progression and presenting a novel therapeutic target for this type of cancer [27]. Sorafenib has also been shown to induce ferroptosis in liver cancer cells [28]. In a clinical trial in patients with diabetes and cardiovascular disease, ferritinophagy induced by bloodletting led to the release of REDOX active iron, aggravating diabetes and cardiovascular disease. In addition, ferritinophagy is involved in the accumulation of free iron associated with the induction of ferroptosis in the pathogenesis of COPD [29]. Emerging evidence suggests that ferritinophagy and ferroptosis play significant roles in cardiovascular diseases [30, 31]. Do Van et al. were the first to highlight the role of ferroptosis in Parkinson's disease development through the activation of protein kinase Cα. Additionally, mice deficient in GPX4 exhibited significant neurological deficits, which could be improved with ferroptosis inhibitors [32]. Ferritinophagy not only plays a crucial role in the pathogenesis of diseases [33] but also contributes to the regulation of efficient erythrocyte production under physiological conditions [34, 35]. Excessive ferritinophagy results in the continued accumulation of iron within the cell, leading to detrimental effects on erythrocyte production. Given that ferritinophagy links iron homeostasis with erythrocyte production, it is reasonable to hypothesize that dysregulation ferritinophagy may contribute to ineffective erythropoiesis of MDS.

When our bodies are exposed to excess iron, they can use cage‐like ferritin to fight iron toxicity. Ferritin is one of the first proteins discovered to play a role in iron metabolism, allowing high concentrations of iron to be stored, thereby preventing exposure to substrates that produce ROS. Each ferritin molecule consists of 24 subunits that can store up to 4500 Fe^3+^ ions [36]. Our study demonstrates that the reduction of the FTH1 level is associated with an increase in ferroptosis, consistent with previous findings of decreased FTH1 levels following induction of ferroptosis through ferritinophagy [37, 38, 39]. However, conflicting studies have shown that elevated FTH1 levels can also lead to increased ferroptosis possibly due to the upregulation of endogenous FTH1 expression in response to cellular iron overload [40]. Furthermore, research on the peroxidation of polyunsaturated fatty acids has indicated an increase in FTH1 expression during ferroptosis [41]. Additionally, overexpression of FTH1 after erastin‐induced ferroptosis has been found to enhance ferritin degradation [42]. Notably, the significant increase in FTH1 protein levels observed following treatment with erastin suggests a role for autophagy‐mediated ferritin degradation [6]. During the early initiation phase, autophagy‐driven ferroptosis generally takes place. Given the complex involvement of autophagy in cell fate determination, it is plausible that FTH1, as a key player in ferritinophagy, may exert distinct effects at different stages of disease progression. Collectively, these findings position FTH1 as a pivotal link between ferroptosis and ferritinophagy in pathological conditions.

Xiuli An and colleagues have documented the significant involvement of autophagy in the process of human erythroid development [43]. We observed a decrease in the expression of GlycoA and impaired haemoglobin formation following FTH1 knockout in K562 cells, These results suggest that FTH1 knockdown may impact erythroid differentiation and haemoglobin synthesis. Our next objective is to validate these observations through animal studies.

Our experiment also has its shortcomings. Sorting samples using more current methods would enable comparing similar stages of differentiation between normal and MDS [44]. We did not differentiate between various developmental stages of nucleated red cells. Furthermore, our study lacks in vivo animal experimental data to substantiate these findings.

In conclusion, the process of ferritinophagy involving FTH1 presents a novel concept for the primary mechanism underlying ineffective haematopoiesis in MDS and suggests that FTH1 may emerge as a promising therapeutic target for MDS‐related anaemia. Moving forward, we aim to utilise cord blood haematopoietic stem cells to silence *FTH1* expression and induce erythroid differentiation, in order to investigate the impact of FTH1 on erythroid differentiation stage and validate our findings through animal experiments.

## Author Contributions

**Liyan Yang:** methodology (equal), writing – original draft (equal). **Mengying Zhang:** methodology (equal), writing – original draft (equal). **Mengyuan Liu:** methodology (equal), writing – original draft (equal). **Yating Yu:** formal analysis (equal), writing – original draft (equal). **Yue Zhang:** methodology (equal), writing – original draft (equal). **Jinyue Yang:** methodology (equal), writing – original draft (equal). **Limin Xing:** formal analysis (equal), writing – original draft (equal). **Zonghong Shao:** project administration (lead), supervision (lead). **Huaquan Wang:** project administration (equal), writing – review and editing (equal).

## Ethics Statement

The study was performed according to the Declaration of Helsinki and approved by the Ethics Committee of Tianjin Medical University General Hospital.

## Consent

Informed consent was obtained from all subjects involved in the study.

## Conflicts of Interest

The authors declare no conflicts of interest.

## Supporting information


**Figure S1.** The sorting purity of GlycoA in MDS patients.
**Figure S2.** Increased ferroptosis and ferritinophagy levels in bone marrow terminally differentiated nucleated erythrocytes were associated with anaemia in MDS patients.
**Figure S3.** The effectiveness of FTH1 knockdown was evidenced through fluorescence microscopy (A), real‐time quantitative transcriptase‐polymerase chain reaction (B), and western blot analysis (C).

## Data Availability

The data used to support the findings of this study are included within the article.
