# Supplementary material for: Loss of FTH1 Induces Ferritinophagy‐Mediated Ferroptosis in Anaemia of Myelodysplastic Syndromes
Source: J Cell Mol Med. 2025 Jan 13;29(1):e70350. doi: 10.1111/jcmm.70350 (PMC11726652; doi:10.1111/jcmm.70350)
Supplement: Supplementary file 1 — Figure S1. The sorting purity of GlycoA in MDS patients. Figure S2. Increased ferroptosis and ferritinophagy levels in bone marrow terminally differentiated nucleated erythrocytes were associated with anaemia in MDS patients. Figure S3. The effectiveness of FTH1 knockdown was evidenced through fluorescence microscopy (A), real‐time quantitative transcriptase‐polymerase chain reaction (B), and western blot analysis (C). [file JCMM-29-e70350-s001.docx]

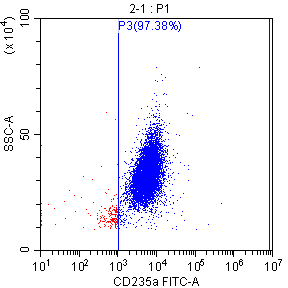


Supplemental Figure 1 The sorting purity of GlycoA in MDS patients.


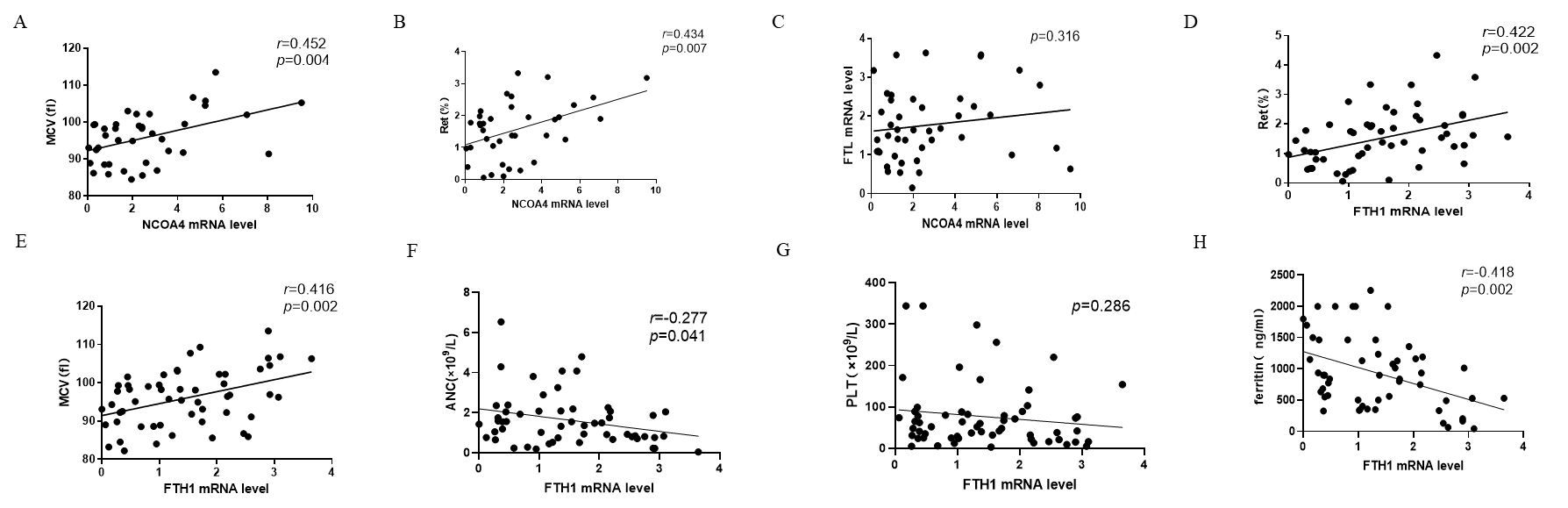


Supplemental Figure 2 Increased ferroptosis and ferritinophagy levels in bone marrow terminally differentiated nucleated erythrocytes were associated with anemia in MDS patients. (A-C) Correlation analysis of *NCOA4* mRNA levels and mean corpuscular volume (MCV) (A), reticulocytes (Ret) percentage (B), *FTL* mRNA levels (C). (D-H) Correlation analysis of *FTH1* mRNA levels and Ret percentage (D), MCV (E), absolute neutrophil counts (ANC) (F), platelets (PLT) (G), ferritin(H).


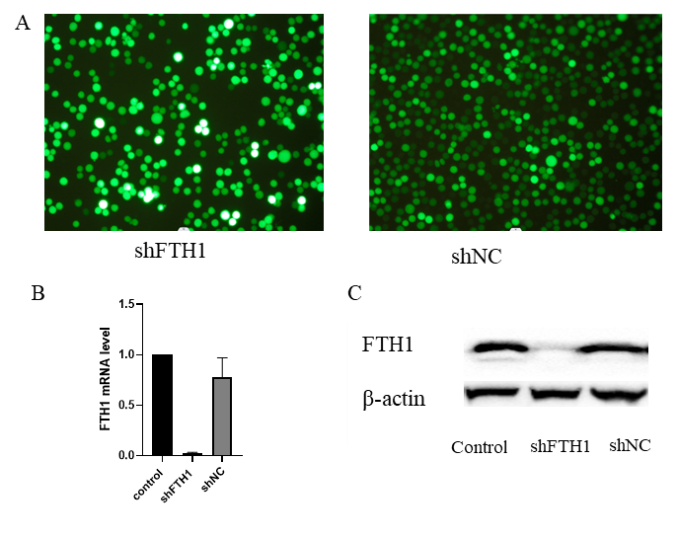


Supplemental Figure 3 The effectiveness of FTH1 knockdown was evidenced through fluorescence microscopy (A), real-time quantitative transcriptase-polymerase chain reaction (B), and western blot analysis (C). Data are presented as mean ± SD for (B).
